# Comparison of prognosis among patients with colorectal cancer liver metastases treated by surgical resection, radiofrequency ablation and HIFU: A protocol for network meta-analysis

**DOI:** 10.1097/MD.0000000000027915

**Published:** 2022-08-19

**Authors:** Li Zhang, Lijuan Qiao, Minghua Zhang, Ya’e Xue, Xueting Zhang, Xiang Gao

**Affiliations:** a Evidence-Based Nursing Center, School of Nursing, Lanzhou University, Gansu, China; b Department of Peripheral Interventional Surgery, Affiliated Hospital of Gansu University of Traditional Chinese Medicine, Gansu, China; c Gansu Provincial Maternity and Child-Care Hospital, Gansu Provincial Central Hospital, Gansu, China; d Gansu University of Chinese Medicine, Lanzhou, China; e Gansu University of Chinese Medicine, Department of Peripheral Interventional Surgery, Affiliated Hospital of Gansu University of Traditional Chinese Medicine, Gansu, China.

**Keywords:** colorectal cancer, liver metastases, network meta-analysis, treat

## Abstract

**Background:**

Colorectal cancer is a malignant tumor second only to lung and breast cancer in the West. The liver is the main target organ for colorectal cancer metastasis, affecting the prognosis and survival. Surgical treatment has made great progress in colorectal cancer liver metastasis, including radiofrequency ablation (RFA), high-intensity focused ultrasound (HIFU) ablation.

**Object:**

Clinical treatments for colorectal cancer liver metastases are not the same. In order to clarify the impact of surgical resection, RFA and HIFU, we provided a decision-making basis for the clinical treatment of colon cancer liver metastasis through systematic reviews and network meta-analysis (NMA).

**Methods:**

We systematically searched the Chinese and English databases: PubMed, Embase, CENTRAL, CINAHL, Web of Science, CNKI, CBM, VIP, Wan Fang. Literature screening, data extraction, and quality evaluation were carried out by two researchers, and finally, use Stata to carry out meta-analysis.

**Results:**

This study is ongoing and the results will be submitted to a peer-reviewed journal for publication.

**Protocol registration number:**

INPLASY202150044.

## 1. Introduction

Colorectal cancer (CRC) is a malignant tumor second only to lung and breast cancer, and the third leading cause of cancer death in the world.^[1,2]^ The mortality rate of CRC cannot be ignored, accounting for 10% of all cancer cases and deaths worldwide.^[3]^ It can be transferred to the liver through blood transfer and abdominal implantation, causing multiple metastatic cancers in the liver.^[4]^ The survey shows that about 25% to 30% of CRCs have liver metastases during the course of the disease.^[5,6]^ When colorectal cancer liver metastases (CRLM) are found, more than 80% of patients with liver metastases cannot be treated with radical surgery, and the five-year survival rate of patients is close to zero.

Surgery is the core treatment for colorectal cancer liver metastasis. Patients with colorectal cancer liver metastases can significantly prolong their survival period. The five-year survival rate can reach 50%, and 25% of patients can be cured. Survival rate over ten years.^[7]^ Hepatic resection is the main treatment, but postoperative recurrence and metastasis seriously affect the survival of patients. Adjuvant chemotherapy, as the main treatment after surgery, can reduce the risk of recurrence and metastasis.^[8,9]^ However, not all patients with liver metastases from colorectal cancer are suitable for surgery. Some patients cannot be operated on due to the large number of liver metastases, scattered distribution, extrahepatic metastases, or physical conditions. At present, the treatment of liver metastases is in addition to surgery, radiotherapy and chemotherapy. In addition, RFA is increasingly used in the treatment of CRLM patients.^[10]^ Local treatment methods represented by RFA play an important role in the treatment of these patients. RFA is a minimally invasive treatment, usually under the guidance of ultrasound, CT or MRI, the electrode is directly placed in the target tissue to kill the tumor. It is a kind of thermal ablation and is the most widely used.^[11]^ The principle of RFA is to rely on various technical means to transmit energy to the tumor site, increase or decrease the local temperature, and kill tissue cells.^[12]^ HIFU is a relatively new technique that has great potential for further development.^[13]^ HIFU is a high-intensity focused ultrasound tumor treatment system. It is a new non-invasive tumor treatment technology in recent years. The small focal area and high energy of HIFU make tumor tissues instantaneously produce coagulation necrosis, which is non-invasive to tumor lesions. One-time complete resection without injury.

Most of the basis for clinical decision-making comes from guidelines or systematic reviews.^[14]^ Network meta-analysis (NMA) is considered the best quality evidence to provide sufficient information for practice.^[15]^ Clinical treatments for colorectal cancer liver metastases are not the same. In order to clarify the impact of surgical resection, RFA and HIFU, we provided a decision-making basis for the clinical treatment of colon cancer liver metastasis through systematic reviews and NMA.

## 2. Methods

### 2.1. Study registration

This NMA has been registered on the International Platform of Registered Systematic Review and Meta-analysis Protocols (INPLASY). The registration number is INPLASY202150044, the DOI number is 10.37766/inplasy2021.5.0044.

### 2.2. Study inclusion and exclusion criteria

#### 2.2.1. Types of studies.

Inclusion: Randomized Controlled Trial without restriction on the use of blind methods.

Exclusion:

1. Non-Chinese and English literature;
2. Incomplete or missing research data;
3. Unable to obtain original documents;
4. Repeated publication of literature;
5. Editorials;
6. Commentaries.

#### 2.2.2. Types of participants.

For patients diagnosed with CRLM, there are no restrictions on gender, age, primary site, primary tumor grade, liver metastasis site, and number of metastases.

#### 2.2.3. Types of interventions.

The patients receive one of the three treatments of HR, RFA and HIFU.

#### 2.2.4. Types of outcomes measures.

Main outcomes:

1. The occurrence of complication (lung infection, incision infection, hemorrhage from liver section)
2. Estimated blood loss
3. The occurrence of relapse (local recurrence, intra-hepatic recurrence, extra-hepatic recurrence)
4. Overall survival rate.

Additional outcomes: Length of hospital stays.

### 2.3. Search strategy

#### 2.3.1. Electronic searches.

We will search the following English electronic bibliographic databases: PubMed (inception-present), Embase (inception-present), Cochrane Central Register of Controlled Trials (CENTRAL) (inception-present), CINAHL (inception-present), Web of Science (inception-present), as well as the Chinese databases: China Knowledge Network (CNKI) (inception-present), China Biomedical Literature Database(CBM) (inception-present), VIP Data(inception-present), Wan Fang Data(inception-present).

#### 2.3.2. Other resources.

Furthermore, reference lists of included RCTs and relevant systematic reviews will be searched. There will be no restrictions on publication year.

#### 2.3.3. Search strategies.

All databases will be based on the MeSH and text word search and will be adjusted according to the specific database. The keywords were as follows: colorectal cancer (“colorectal cancer” OR “colorectal carcinoma” OR “rectal cancer” OR “rectal carcinoma” OR “colon cancer” OR “colorectal cancer” OR “colorectal carcinoma” OR “carcinoma of colon” OR “colorectal neoplasms”).

#### 2.3.4. Literature screening.

All search results are imported into ENDNOTE X8 literature management software, eliminate repetitive literature after reading the title and first rule out the obvious is not in conformity with the requirements of documents, read the full text of the literature of remaining, clear whether conform to its standard, if necessary, through a variety of contact information (phone, email) to contact the author to obtain the required information, to be incorporated into the literature by the two researchers (LZ, LJQ) cross check whether accord with a standard, For the literature with uncertain inclusion, the decision was made after careful reading by the third researcher (LZ).

#### 2.3.5. Data extraction.

After careful reading of the included literature, we will use Microsoft Excel 2013 to create a pre-determined data extraction table to collect relevant information and data:

1. Basic information: article title, first author, publication time, country/region, etc.;
2. Research characteristics: intervention measures of the experimental group and control group, number of subjects, age, original location and transfer method;
3. Key information needed for literature bias risk evaluation;
4. Required outcome indicators. The data will be extracted independently by two reviewers (LJQ, XG). Any differences will be settled through discussions between the two reviewers or by the third researcher (XTZ).

### 2.4. Study quality assessment

The methodological quality of the final included RCT will be evaluated independently by two reviewers (LJQ, XG). Any disagreements will be resolved through discussion between the two parties or decided by a third reviewer (LZ). The research quality of RCTs was evaluated by two researchers using the tools recommended by Cochrane System Reviewer Manual 5.1 to assess the risk of bias,^[16,17]^ and Rev Man 5.3 was used to draw the risk of bias related chart. This tool includes random methods, allocation hiding, blinding (researcher and subject), blinding (outcome measurer), complete outcome data, selective reporting of results, and other sources of bias. Each aspect can be further classified as low risk, high risk or unclear risk.

### 2.5. Statistical analysis

#### 2.5.1. Data synthesis.

In this study, Stata software was used for data analysis and comparison, and relative risk and 95% confidence interval (95% CI) were used as the analysis statistics of binary variables. Use inconsistency test to detect whether there is inconsistency between direct evidence and indirect evidence. The inconsistency test was performed by node analysis, and if *P* > .05, the consistency model was used for analysis. At the same time, the node splitting method is used to check the local inconsistency. When direct evidence and indirect evidence are inconsistent, use RevMan 5.3 for direct comparison. If *P* > .05, it is considered that there is no overall inconsistency; if the 95% CI of the ROR contains 1, it is considered that there is no local inconsistency, otherwise there is local inconsistency.^[18]^ Heterogeneity is judged by the prediction interval graph. If the 95% CI and 95% prediction interval (95% Pr I) both contain one or both do not contain 1, then it is considered that there is no statistical heterogeneity, otherwise, there is statistical heterogeneity. By calculating the area evaluation under the cumulative ranking curve (SUCRA).^[19]^

#### 2.5.2. Subgroup analysis.

If the evidence is sufficient, we will conduct a subgroup analysis to determine the differences between different genders, ages, primary sites, primary tumor grades, and metastasis methods.

### 2.6. Quality of evidence

Two reviewers (YSZ, ZBZ) will use the GRADE (Grading of Recommendations Assessment, Development and Evaluation) method to assess the quality of evidence of included studies. GRADE contains five domains, including bias risk, consistency, directness, precision, and publication bias. The evidence levels classified into four levels: high, moderate, low, or very low.

### 2.7. Sensitivity analysis

Exclude low-quality studies to complete sensitivity analysis.

### 2.8. Summary of findings

A “summary of finding” table will be created for the major outcome. We will also add absolute and relative percentage changes to the “summary of finding”’. For detailed information, see Table 1; We have listed partial summary of findings for the main comparison.

**Table 1:** Summary of findings for the main comparison.

HF compared with RFA setting						
Intervention: HF						
Comparison: RFA						
	Illustrative comparative risks* (95% CI)		Relative effect (95% CI)	No of participants (studies)	Quality of the evidence (GRADE)	Comments
Outcome	Assumed risk	Corresponding risk				
	HF	RFA				
Lung infection						
Incision infection						
Hemorrhage from liver section						
Estimated blood loss						
The occurrence of relapse						
Overall survival rate						

HIFU = high-intensity focused ultrasound, HR = hepatic resection, RFA = radiofrequency ablation.

## 3. Result

**Figure 1.**
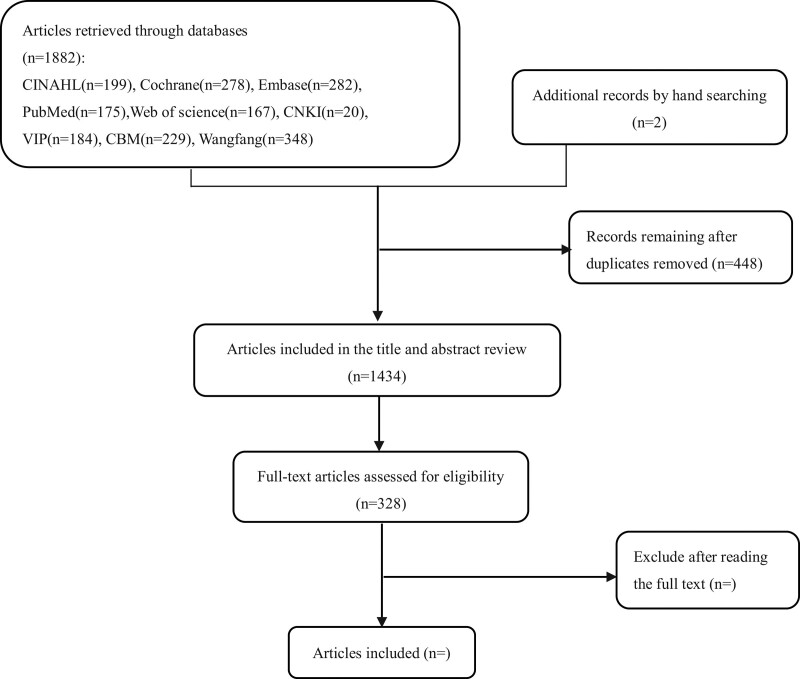
Summary of evidence search and selection.

We identified 1882 records through database searching and two records through other sources. The detailed search flowchart is shown in Figure 1.

## 4. Discussion

Liver metastasis of colon cancer is one of the key and difficult points in the treatment of colon cancer.^[20]^ According to statistics, liver metastasis has been observed in more than 25% of patients when colon cancer is clearly diagnosed. After removal of the primary tumor, up to 25% of patients have liver metastases. During the entire tumor treatment process, about 50% of colon cancer patients may develop liver metastases.^[21]^ Neoadjuvant chemotherapy can prolong the survival of patients and provide radical surgery opportunities for patients with advanced colon cancer.^[22]^ Faced with such a large population, there is currently no uniform standard for the selection of treatment options for colon cancer liver metastasis, and there is still a lack of large-scale prospective, randomized, and controlled clinical studies to compare the survival rates of various treatment options. Different treatment options have a greater impact on the prognosis of patients. In order to clarify the impact of surgery, RFA and chemotherapy on the survival rate of patients, this study conducted a systematic review and a NMA. There are some limitations in this study. Firstly, due to the limitations of English and Chinese, there may be some risk of bias.

## Author contributions

Conceptualization: Xiang Gao, Ya’e Xue.

Methodology: Lijuan Qiao, Xueting Zhang.

Software: Li Zhang, Lijuan Qiao, Xueting Zhang.

Writing-original draft: Li zhang, Minghua Zhang, Ya’e Xue.

Writing-review & editing: Li Zhang, Lijuan Qiao, Xiang Gao
